# Experimental Study on Tensile Properties of a Novel Porous Metal Fiber/Powder Sintered Composite Sheet

**DOI:** 10.3390/ma9090712

**Published:** 2016-08-23

**Authors:** Shuiping Zou, Zhenping Wan, Longsheng Lu, Yong Tang

**Affiliations:** School of Mechanical and Automotive Engineering, South China University of Technology, Guangzhou 510640, China; meshpzou@mail.scut.edu.cn (S.Z.); meluls@scut.edu.cn (L.L.); ytang@scut.edu.cn (Y.T.)

**Keywords:** porous metal composite, porous metal fiber sintered sheet, porosity, tensile properties

## Abstract

A novel porous metal fiber/powder sintered composite sheet (PMFPSCS) is developed by sintering a mixture of a porous metal fiber sintered sheet (PMFSS) and copper powders with particles of a spherical shape. The characteristics of the PMFPSCS including its microstructure, sintering density and porosity are investigated. A uniaxial tensile test is carried out to study the tensile behaviors of the PMFPSCS. The deformation and failure mechanisms of the PMFSCS are discussed. Experimental results show that the PMFPSCS successively experiences an elastic stage, hardening stage, and fracture stage under tension. The tensile strength of the PMFPSCS is determined by a reticulated skeleton of fibers and reinforcement of copper powders. With the porosity of the PMFSS increasing, the tensile strength of the PMFPSCS decreases, whereas the reinforcement of copper powders increases. At the elastic stage, the structural elastic deformation is dominant, and at the hardening stage, the plastic deformation is composed of the structural deformation and the copper fibers’ plastic deformation. The fracture of the PMFPSCS is mainly caused by the breaking of sintering joints.

## 1. Introduction

Porous metals exhibit high porosity, good capacity for energy absorption, perfect permeability, superior mechanical properties, and excellent thermal and electric conductivity. With these outstanding physical and mechanical properties, they have been widely applied to sound absorption [1], jet noise reduction [2], catalytic applications [3], gas diffusion layers [4], phase change heat transfer, energy absorption [5], intermediate materials for filtration and separation [6] and bioimplants [7]. Currently, porous metals mainly include porous metal powder sintered materials [8], porous metal fiber/mesh sintered sheets [9], metal foams [10] and entangled metal wire materials [11]. However, the porous metals with a single porous structure cannot adequately meet some of the special requirements of expanded applications. Recently, some composite porous metals have been developed by different methods, and the relationships between the mechanical properties and the structure of composite porous metals have attracted well-deserved attention. Jiang et al. [12] produced a graded porous titanium-magnesium composite via infiltration casting and acid etching, and the compression properties of this porous metal composite were investigated. Ma et al. [13] fabricated the porous Cu–Sn–Ti metal with alumina bubble particles, and the mechanical strength of the porous Cu–Sn–Ti metal was researched. Cay et al. [14] synthesized a porous alumina-reinforced Mg composite through a powder metallurgical method and investigated the mechanical behavior using compression testing. Rabiei et al. [15] produced an aluminum–steel composite metal foam via casting, and the modules of elasticity [16], monotonic compression properties [17] and energy absorption capability [18] of this composite foam were also studied. Tang et al. [19] developed a sintered porous metal composite made of copper foam and copper powders, and the tensile properties of this sintered porous metal composite were investigated. Ackermann et al. [20] synthesized a dual-scale reticulated porous ceramic using the foam replication method, and the morphological properties and effective thermal conductivity were determined in their work.

In this study, a novel porous metal fiber/powder sintered composite sheet (PMFPSCS) is developed by solid-state sintering of a mixture of the PMFSS and copper powder to obtain a composite porous structure. The PMFSS acts as a three-dimensional porous skeleton and copper powders with a spherical shape are used as filling materials. The microstructure of this PMFPSCS is observed by scanning electron microscopy (SEM) and the sintering density and porosity of the PMFPSCS are determined. The uniaxial tensile behavior and mechanical properties of the PMFPSCS are investigated and the deformation and failure mechanisms are discussed based on experimental observations.

## 2. Results

### 2.1. Structure Characterization of the PMFPSCS

**Figure 1:**
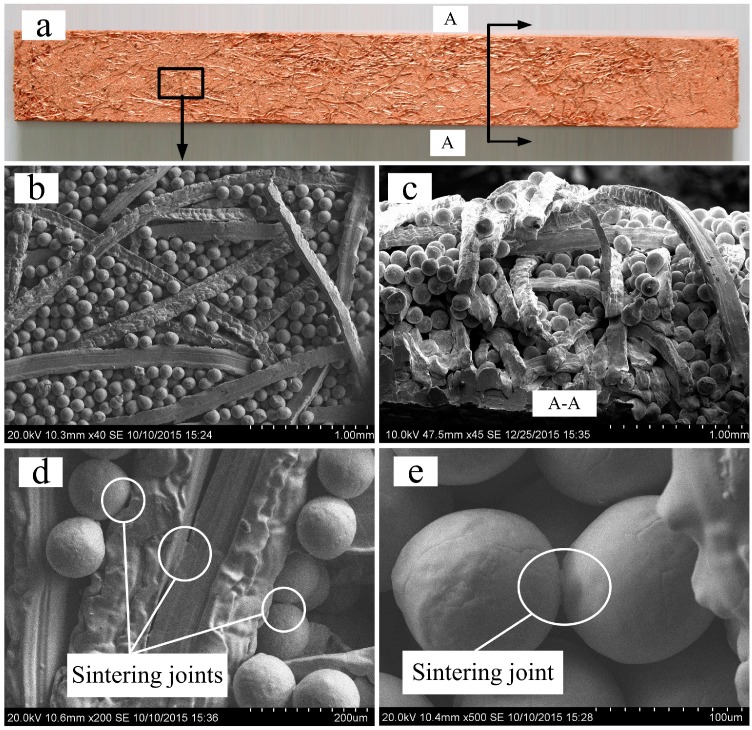
Characteristics of the PMFPSCS: (**a**) appearance of PMFPSCS; (**b**) SEM image of PMFPSCS; (**c**) cross section of PMFPSCS; and (**d**,**e**) sintering joints of PMFPSCS.

The characteristics of PMFPSCS sintered at 900 °C for 60 min are shown in Figure 1 in which the porosity of the PMFSS skeleton is 80% and the diameter of copper particles is between 75 and 100 μm. The PMFPSCS consists of a three-dimensional reticulated skeleton, filled copper powders and irregular micropores. Looking at the longitudinal section and cross section of the sample, a uniform copper powders distribution is visible, as shown in Figure 1b,c, and the amount of copper powders is the same statistically in the upper and bottom part of the sample. The pore wall can be fibers, powders and intercrossing conjunctive points, which exhibit a complex and through-connected porous structure. The intercrossing conjunctive points may become joint points after being sintered as shown in Figure 1d,e. Since there is no distinct difference in the pore distribution, the PMFPSCS prepared in this study is homogenous, which ensures the materials are isotropic and the measurements reproducible. Obviously, this kind of porous composite materials combines the advantages of porous metal fiber sintered sheets and porous metal powder sintered materials. Therefore, the PMFPSCS is a promising capillary wick due to its composite porous structure, which possesses high capillary force as well as high permeability.

**Figure 2:**
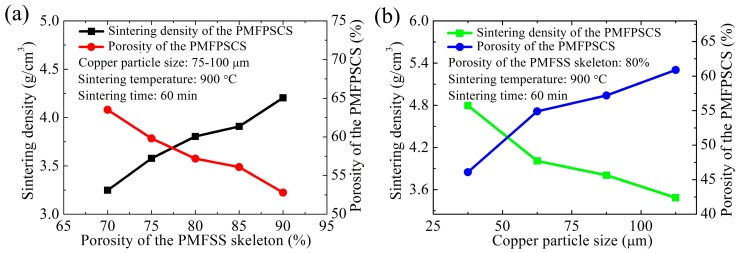
Sintering density and porosity of the PMFPSCS versus different structure parameters sintered at 900 °C for 60 min: (**a**) different porosities of the PMFSS skeleton; and (**b**) different copper particle sizes.

The sintering density and porosity of the PMFPSCS versus the porosity of the PMFSS skeleton and the copper particle size are shown as Figure 2 where the PMFPSCS is prepared at a sintering temperature of 900 °C for 60 min. From Figure 2a, it can be found that the sintered density of the PMFPSCS is 3.25 g/cm^3^ and the porosity of the PMFPSCS is 63.5% when the porosity of the PMFSS skeleton is 70%; whereas the sintered density increases to 4.21 g/cm^3^ and the porosity decreases to 52.8% when the porosity of the PMFSS skeleton increases to 90%. With higher porosity of the PMFSS skeleton, there is much more space per unit volume for the loose packing of copper particles. In this way, the mass of the PMFPSCS with a higher porosity of the PMFSS skeleton is larger, leading to a larger sintering density but a lower porosity of the PMFPSCS. Figure 2b shows the sintering density of the PMFPSCS decreases while the porosity of the PMFPSCS increases with the increase in size of the copper particles. Smaller copper particles more easily fill the three-dimensional porous structure of the PMFSS skeleton. In this case, more copper particles are filled into the PMFSS skeleton with the same porosity, which leads to a larger sintering density but lower porosity of the PMFPSCS.

It is worth pointing out that, unlike porous copper powder sintered materials, the sintering density and porosity of the PMFPSCS prepared at different sintering temperatures and sintering times are almost constant. Though the copper particles inside the PMFSS may shrink during the sintering process, the volume of the PMFPSCS changed little due to the support of the PMFSS skeleton. Therefore, the sintering temperature and sintering time have minimal effect on the sintering density and porosity of the PMFPSCS.

### 2.2. Uniaxial Tensile Behavior of the PMFPSCS

**Figure 3:**
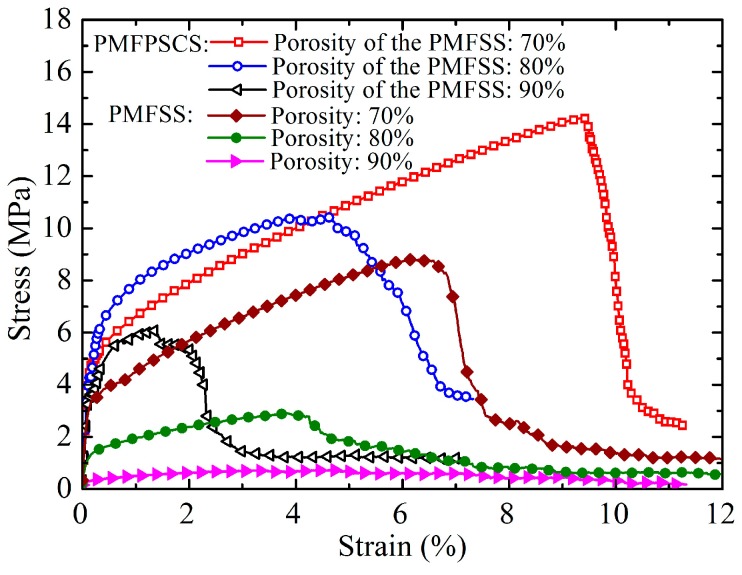
Tensile stress–strain plots of the PMFPSCS with a copper size of 75–100 μm and PMFSS with different porosities sintered at 900 °C for 60 min.

**Figure 4:**
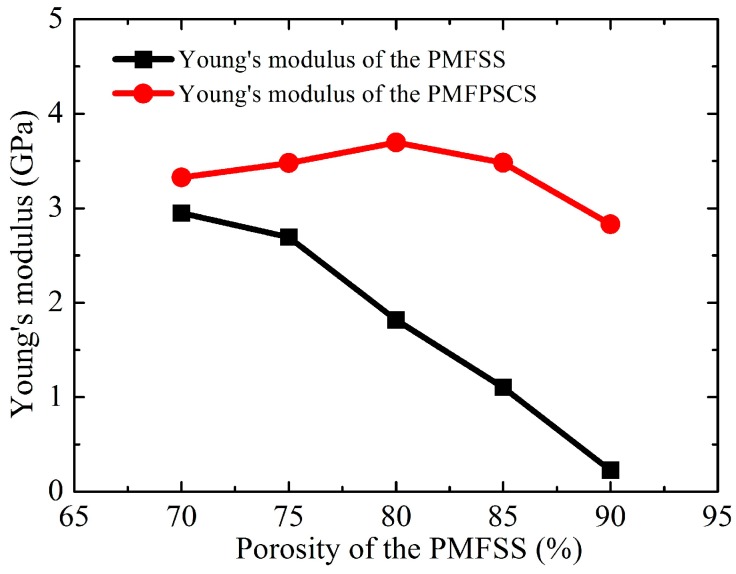
The Young’s modulus of the PMFPSCS and PMFSS.

The typical uniaxial tensile stress–strain plots of the PMFPSCS sintered at 900 °C for 60 min are shown in Figure 3 where the PMFPSCSs are made of copper particles with 75–100 μm in diameter and a PMFSS skeleton with a porosity of 70%, 80% and 90%, respectively. Correspondingly, the amounts of copper powders mixed are 3.08 g, 7.29 g, and 11.93 g. It can be seen from Figure 3 that with the increase of strain, the PMFPSCS specimen successively experiences an elastics stage, hardening stage and fracture stage. Compared with other stages, the elastic stage is very short and the strain is only about 0.05%–0.08%. At the initial elastic stage, the stress–strain relationship meets the linear elastic Hooke’s law. The Young’s modulus can be estimated from the linear stress–strain relationship as shown in Figure 4, in which the Young’s modulus of the PMFPSCS is larger than that of the PMFSS. In addition, by increasing the porosity of the PMFSS, the Young’s modulus of the PMFPSCS increases with a very small slope if the porosity of the PMFSS is below 80%, henceforth decreasing with a large slope. After the elastic stage, a long hardening stage follows, which indicates a complex stress–strain progression. The hardening stage depends on the porosity of the PMFSS, and the strength and elongation decrease with an increasing in the porosity of the PMFSS. When the porosity of the PMFSS is 80%, the strength and elongation of the PMFPSCS are 10.37 MPa and 4.67%; whereas, when the porosity of the PMFSS is 90%, the strength and elongation of the PMFPSCS are reduced to 6.09 MPa and 1.36%, respectively. However, compared with the PMFSS, the strength and elongation of the PMFPSCS increase significantly due to the mixing of the copper powders. When the porosity of the PMFSS is 80%, its strength and elongation are only 2.92 MPa and 3.80%, and the strength and elongation of the PMFSS with a porosity of 90% are 0.72 MPa and 3.12%, respectively. The larger the porosity of the PMFSS skeleton, the greater is the reinforcement effect of the mixed copper powders. After the stress reached the maximum value, the PMFPSCS sample begins to fracture. This process can be rapid because the fibers and sintering joints have already experienced a large deformation and are at their ultimate stress level. The rapid fracture causes a sharp drop in stress as shown in Figure 3.

### 2.3. Effect of the Structural Parameters on the Tensile Properties of the PMFPSCS

The experiments suggest that the uniaxial tensile properties of the PMFPSCS depend significantly on the porosity of the PMFSS and the amounts of mixed copper powder. Revealing the relationship between the uniaxial tensile properties and the structural parameters is important when tailoring porous structures and mechanical properties.

**Figure 5:**
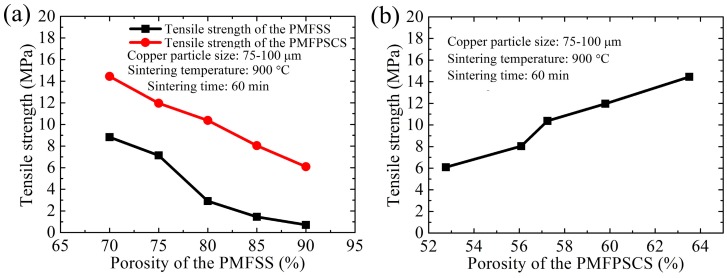
Tensile strength of the PMFPSCS: (**a**) tensile strength of the PMFPSCS and PMFSS vs. porosity of the PMFSS; and (**b**) tensile strength of the PMFPSCS vs. porosity of the PMFPSCS.

The influence of porosity of the PMFSS on the uniaxial tensile properties of the PMFPSCS is investigated using specimens with 70%, 75%, 80%, 85% and 90% porosity of the PMFSS skeleton. Figure 5 shows the uniaxial tensile strength of the PMFPSCS sintered at 900 °C for 60 min with a copper particle size of 75–100 μm, while the amounts of mixed copper powders are 3.08 g, 4.87 g, 7.29 g, 9.26 g and 11.93 g, respectively. From Figure 5, it can be found that the tensile strength of the PMFPSCS ranges from 14.45 MPa to 6.09 MPa. Thus, the uniaxial tensile strength of a PMFPSCS depends significantly on the porosity of the PMFSS skeleton and decreases dramatically when the porosity of the PMFSS increases. When the copper particle size remains unchanged, the amount of mixed copper powder increases with an increase in the porosity of the PMFSS skeleton, resulting in a decrease in the porosity of the PMFPSCS. Therefore, the uniaxial tensile strength of a PMFPSCS increases with an increase in the porosity of the PMFPSCS in this case, as shown in Figure 5b. In addition, from Figure 5a, it can also been seen that the tensile strength of the PMFPSCS is much larger than that of the PMFSS due to the reinforcement of copper powders.

**Figure 6:**
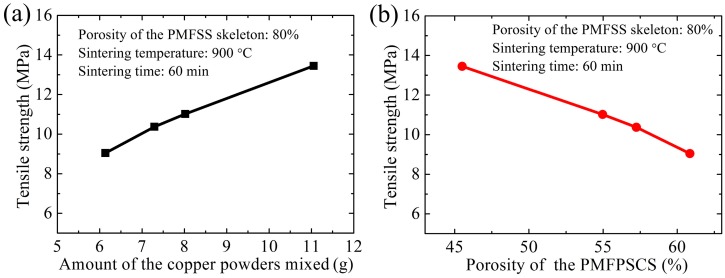
Tensile strength of the PMFPSCS: (**a**) tensile strength vs. the amount of the mixed copper powders; and (**b**) tensile strength vs. the porosity of the PMFPSCS.

As another important structural parameter of the PMFPSCS, the amount of mixed copper powders can also affect the tensile strength of the PMFPSCS. The specimens are sintered at 900 °C for 60 min with copper particle sizes of 25–50 μm, 50–75 μm, 75–100 μm and 100–125 μm, while the amounts of mixed copper are 11.05 g, 8.02 g, 7.29 g and 6.13 g, respectively. In the specimens, the porosity of the PMFSS is 80%. Figure 6 shows the influence of the amount of mixed copper powders on the tensile strength the PMFPSCS. From Figure 6, it can be found that the tensile strength of the PMFPSCS increases with an increase in the amount of copper powder when the porosity of the PMFSS remains unchanged. However, with an increase of the amount of copper powder, the porosity of the PMFPSCS decreases. In this case, the uniaxial tensile strength of a PMFPSCS decreases with an increase in the porosity of the PMFPSCS, as shown in Figure 5b.

### 2.4. Effect of the Sintering Parameters on the Tensile Properties of the PMFPSCS

**Figure 7:**
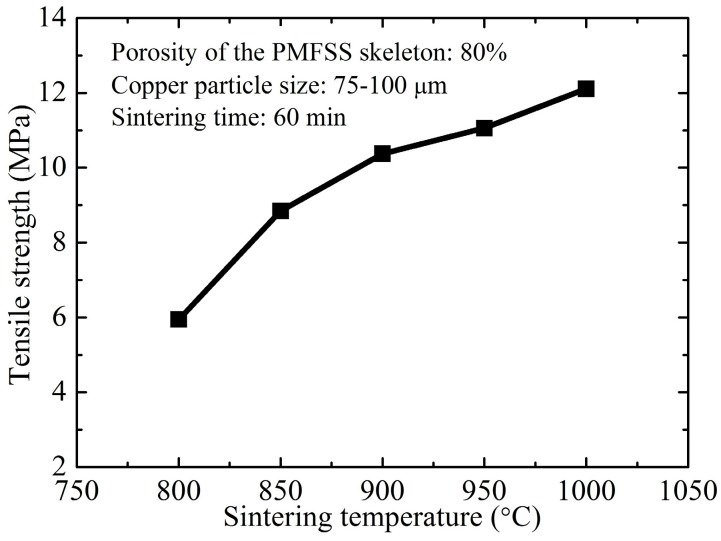
Tensile strength of the PMFPSCS obtained at different sintering temperatures.

The sintering parameters including sintering temperature and sintering time also have a significant influence on the tensile strength of the PMFPSCS. The influence of the sintering temperature on the tensile strength of the PMFPSCS is investigated using the specimens prepared at sintering temperatures of 800 °C, 850 °C, 900 °C, 950 °C and 1000 °C for 60 min. The porosity of the PMFSS skeleton is 80% and the copper particle size is 75–100 μm. Figure 7 shows the influence of the sintering temperature on the tensile strength of the PMFPSCS. From Figure 7, it can be found that the tensile strength of the PMFPSCS is 5.95 MPa when the sintering temperature is 800 °C. However, the tensile strength of the PMFPSCS increased to 12.11 MPa when the sintering temperature is 1000 °C. With the sintering temperature increasing, the velocity of material migration is accelerated, causing the sintering joints to grow more quickly, which is helpful to enhance tensile strength.

**Figure 8:**
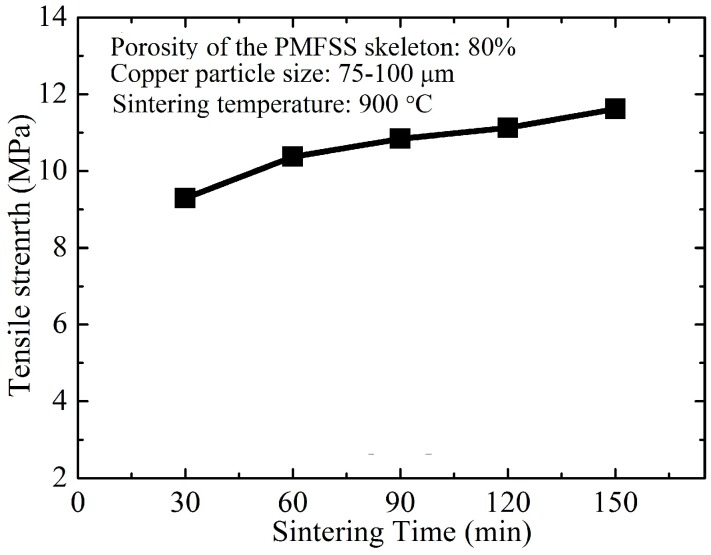
Tensile strength of the PMFPSCS obtained at different sintering times.

Figure 8 presents the relationship between sintering time and tensile strength. In the experiments, the tested specimens with 80% porosity of the PMFSS skeleton and a copper particle size of 70–100 μm are prepared at a sintering temperature of 900 °C for 30 min, 60 min, 90 min, 120 min and 150 min, respectively. From Figure 8, it is found that, depending on the sintering time, the tensile strength of PMFPSCS ranges from 9.30 to 11.62 MPa. The tensile strength of PMFPSCS increases slowly with an increase in sintering time. It is well known that prolonging the sintering time can promote the formation and growth of sintering joints since material in the contact regions has enough time to diffuse, and, therefore, the tensile strength is improved. However, the sintering time has less effect on the increasing of the tensile strength than the sintering temperature.

## 3. Discussion

### 3.1. Deformation and Failure Mechanism of the PMFPSCS

Though the PMFPSCS exhibits some similarities in tension behavior with other PMFSS [8,21] or entangled steel wire material [11,22], the PMFPSCS reveals its own tensile behaviors and deformation mechanisms. The total elastic deformation of the PMFPSCS is caused by the structural elastic deformation, the elastic deformation of fibers and copper powders sintered together. It is difficult to evaluate their exact contributions in the total deformation. However, it can be assumed that the structural elastic deformation is dominant because the elastic modulus of the PMFPSCS (3.70 GPa) is much smaller than that of the red copper (97.80 GPa). The fibers and copper powders sintered together undergo elastic deformation only to coordinate the structural deformation. However, the copper powders sintered inside the PMFSS skeleton become an obstacle to the structural deformation. Therefore, the Young’s modulus of PMFPSCS is larger than that of the PMFSS, as shown in Figure 4.

**Figure 9:**
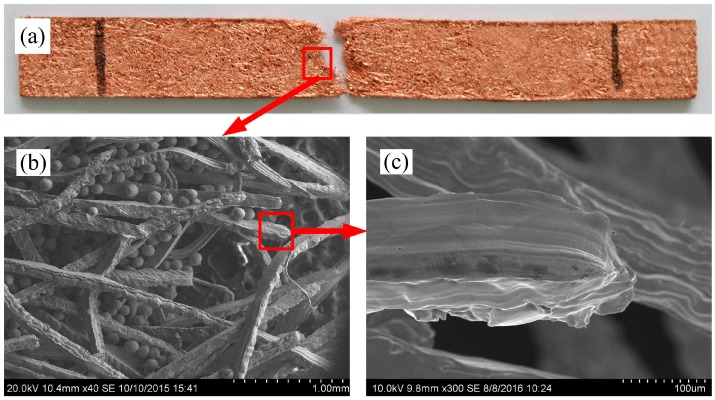
Failed PMFPSCS sample: (**a**) appearance of failed PMFPSCS; (**b**) SEM image of fracture region; and (**c**) SEM image of fractured copper fiber.

As the stress increases, the strain of the PMFPSCS evolves through the structural plastic deformation as well as the copper fiber plastic deformation. The plastic deformation of copper powders inside the PMFSS skeleton can be ignored because the sintered copper powder materials are brittle and frail [19]. The structural plastic deformation causes a more uniform stress distribution along the cross section of the PMFPSCS. Hence, more copper fibers will be involved in further deformation. Higher tensile loading is therefore required to maintain the deformation. Thus, a strain–hardening effect occurs as shown in Figure 3. With the increase of the porosity of the PMFSS, the strain–hardening effect is weakened because the total plastic deformation of the PMFPSCS is composed of the structural plastic deformation and the copper fiber plastic deformation, while the sintered copper powders hinder the plastic deformation. At a late stage of the plastic deformation, some local sintering joints begin to break and a sharp fluctuation appears in the stress–strain curve. The higher the porosity of the PMFSS, the more distinct this fluctuation, as shown in Figure 3. When some local sintering joints are broken, the bearing capacity of the PMFPSCS decreases and the rupture of the PMFPSCS will occur instantaneously, resulting in a sharp drop in stress, as shown in Figure 3. Figure 9 presents the fracture morphologies of the PMFPSCS after the uniaxial tensile test. From Figure 9b, it can be seen that a large amount of copper powder falls off and the reticulated structure becomes loose due to the breaking of the sintering joints. In addition, it can be concluded from Figure 9c that some fibers in the array undergo a necking deformation and ductile fracture. The reason for this is that localized stress reaches the tensile strength of fiber due to stress concentration.

### 3.2. Tensile Properties of the PMFPSCS

**Figure 10:**
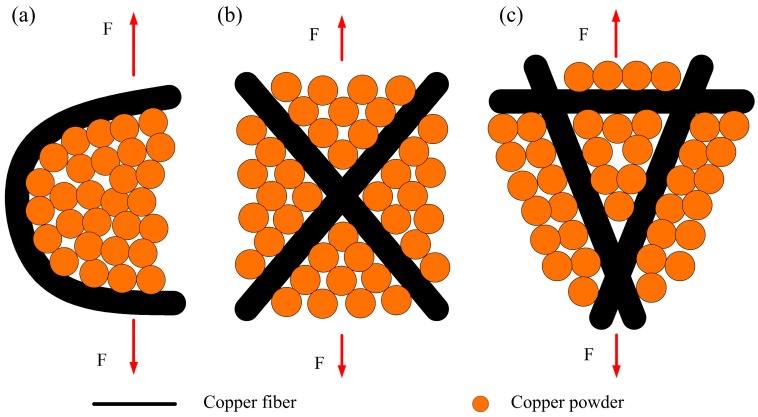
Basic connection modes between fibers and powders in the PMFPSCS: (**a**) intersection connection; (**b**) cross connection; and (**c**) reticulation connection.

Based on the observed characteristics of the PMFPSCS, the copper fibers are randomly intertwined and the copper powders fill in the interspace of the reticulated skeleton. Hence, a three-dimensional reticulated skeleton forms in which large numbers of conjunctions exist and contribute to the strength after sintering. The basic connection modes of the PMFPSCS include intersection connection, cross connection, and reticulation connection, as shown in Figure 10. It can be concluded that the yielding strength and ultimate strength of the PMFPSCS are determined by the reticulated skeleton and reinforcement of copper powders. Consequently, the strength of the PMFPSCS is higher than that of the PMFSS with the same porosity in the PMFSS. With the increase of the PMFSS porosity, the amount of copper fiber decreases and the amount of mixed copper powder increases. Therefore, the strength of the PMFPSCS decreases with an increase in the porosity of PMFSS, and, correspondingly, the reinforcement of copper powders becomes more distinct as shown in Figure 3 and Figure 5. When the porosity of the PMFSS is constant, the amount of copper powder increases with copper particle size decreasing. In this case, the strength of the PMFPSCS increases as shown in Figure 6.

In addition, it is worth pointing out that PMFPSCS of 80% has higher stress than that of 70% at a strain of 0.2%–5% as shown in Figure 3. From Figure 4, it can be found that the Young’s modulus of PMFPSCS of 80% is larger than that of 70%. Accordingly, the yielding strength of the PMFPSCS of 80% is higher than that of 70%. Therefore, at the hardening stage of the PMFPSCS of 80% (with a strain of 0.2%–5%), the PMFPSCS of 80% has higher stress than that of 70%.

## 4. Experimental Procedures

### 4.1. Preparation of the PMFPSCS

The preparation of the PMFPSCS includes five processes: copper fiber manufacturing, segmented copper fiber bedding, pre-sintering, copper powder packing and sintering. During the manufacturing process of copper fiber, a self-designed multi-tooth tool with a row of tiny teeth with triangular cross-sections was used to manufacture the copper fiber [23]. Dry cutting experiments were conducted on precise lathe C6132A (Guangzhou Machine Tool Works, Guangzhou, China). A high-speed steel tool was used to machine the workpiece with a diameter of 50 mm at cutting depth of 0.2 mm, feed of 0.2 mm/r and cutting speed of 16.5 m/min. Subsequently, the copper fiber produced with an equivalent diameter of 100 μm is segmented into short fibers of 15 mm in length. The segmented fibers are bedded in a mold cavity layer by layer, both disorderly and uniformly. After the filling work is accomplished, the packing condition is held by screwing the bolts. Then, the bedded copper fibers are sintered in an oven-type furnace under hydrogen atmosphere. The sintering temperature was set as 800 °C and the sintering time was 60 min. The heating rate was kept at 5 °C/min before the temperature reached the degree that was 50 °C lower than the final sintering temperature; after that, the heating rate was kept at 1.5 °C/min. The pressure of the hydrogen gas inside the furnace chamber was 0.3 MPa. After the sintering process, the specimens were cooled to room temperature in the chamber of the furnace. In this way, the PMFSS acting as the porous skeleton in the PMFPSCS is obtained. Then, the PMFSS was fitted into a mold cavity whose shape and dimension are the same as the PMFSS specimen and copper powders are filled into the PMFSS in a loose packing through ultrasonic vibration. Finally, the semi-fished PMFPSCS is sintered in an oven-type furnace in a hydrogen atmosphere. The sintering temperature was set between 800 °C and 1000 °C while the sintering time ranges from 30 min to 150 min. The sintering process is the same as the pre-sintering process as mentioned previously.

The average porosity of the PMFSS can be calculated by the quality-volume method, as shown in the following equation:

(1) $$E(\%)=(1-\frac{M_{1}}{\rho V})\times 100\%$$

where *E*, *M*_1_ and *V* indicate the porosity, mass (g) and volume (cm^3^) of the copper fibers, respectively. *ρ* is the density (g/cm^3^) of copper.

After packing the copper powder loosely and when final sintering process is completed, the volume of specimen changes little due to the protection of the PMFSS skeleton. Hence, the sintering density of PMFPSCS can be calculated by the following equation:

(2) $$\rho_{s}=\frac{M}{V}$$

where *ρ_s_* is the sintering density and *M* is the mass (g) of the PMFPSCS specimen.

**Figure 11:**
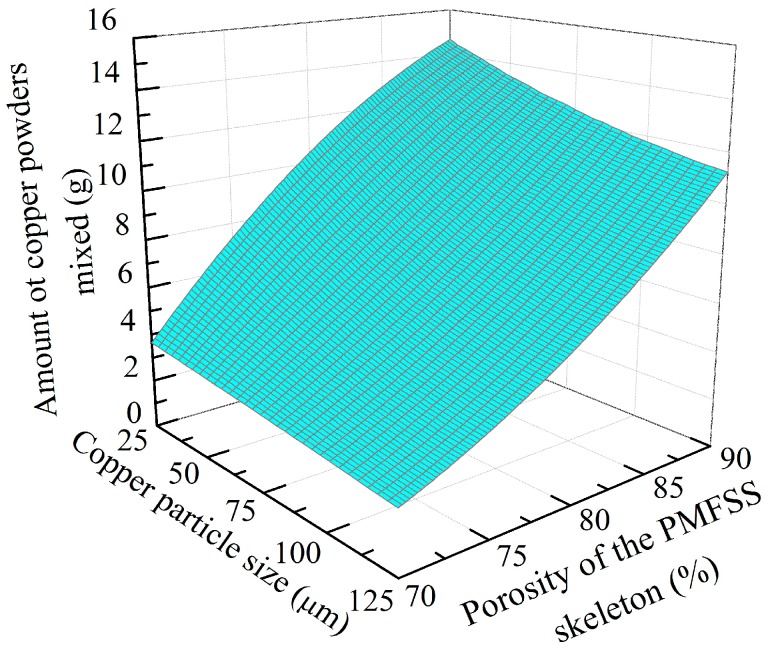
Relationship between the amount of filled copper powder and the porosity of the PMFSS and copper particle size.

The amount of the filled copper powder is determined by the porosity of the PMFSS and copper particle size, and their relationship is shown in Figure 11. From Figure 11, the amount of filled copper powder increases with an increase in the porosity of the PMFSS and decreases with an increase in the size of the copper particles. The effect of the porosity of the PMFSS is more distinct.

The average porosity of the PMFPSCS can be calculated by the quality-volume method, as shown in the following equation:

(3) $$\theta (\%)=(1-\frac{\rho_{s}}{\rho })\times 100\%=(1-\frac{M}{\rho V})\times 100\%$$

### 4.2. Uniaxial Tensile Test of the PMFPSCS

All the tensile tests of PMFPSCS were carried out on a PC-controlled electronic universal testing machine (RGL-20A, Reger Instrument Co., Ltd., Shenzhen, China) at room temperature (approximately 25 °C). The dimensions of the PMFPSCS specimen were 120 mm in length, 15 mm in width and 2 mm in thickness. To avoid the deformation of the PMFPSCS network structure in the clamping process, rubber mats were placed between clamps before tensile testing. After the clamping, the effective length of specimen is 80 mm. The tensile tests of all specimens were performed at a constant speed of 1.5 mm/min using displacement control. Strain is defined as the ratio of the elongation to the initial gauge length of specimen. In order to reduce error, three specimens were tested for each case and an average value was calculated as the tensile strength and specific tensile strength of the PMFPSCS specimen.

## 5. Conclusions

(1) A novel porous metal fiber/powder sintered composite sheet is developed by sintering a mixture of the PMFSS and copper powders. The PMFPSCS consists of a three-dimensional reticulated skeleton of metal fiber, sintered copper powers and irregular and through-connect micropores.

(2) The PMFPSCS displays three-stage stress–strain behavior under uniaxial tension, i.e., an elastic stage, hardening stage and fracture stage. At the initial elastic stage, the structural plastic deformation of the PMFPSCS is dominant, and at the hardening stage, the plastic deformation is caused by structural plastic deformation of the PMFPSCS as well as the plastic deformation of copper fibers. With an increase of the porosity of the PMFSS, the strain–hardening effect becomes weak.

(3) The tensile strength of the PMFPSCS is determined by the tensile strength of the PMFSS and reinforcement of copper powders. With the porosity of the PMFSS increasing, the tensile strength of the PMFPSCS decreases, but the reinforcement of copper powders becomes more conspicuous. The breaking of the sintering joints is the main failure mode of the PMFPSCS.

(4) Both an increase of sintering temperature and sintering time can strengthen the PMFPSCS, whereas sintering temperature has a more significant effect on the strength of the PMFPSCS than sintering time.

## References

[1] Zhang B., Chen T.N. (2009). Calculation of sound absorption characteristics of porous sintered fiber metal. Appl. Acoust..

[2] Tan J.C., Clyne T.W. (2008). Ferrous fibre network materials for jet noise reduction in aeroengines Part II: Thermo-mechanical stability. Adv. Eng. Mater..

[3] Reichelt E., Heddrich M.P., Jahn M., Michaelis A. (2014). Fiber based structured materials for catalytic applications. Appl. Catal. A Gen..

[4] Yi P.Y., Peng L.F., Lai X.M., Li M.T., Ni J. (2012). Investigation of sintered stainless steel fiber felt as gas diffusion layer in proton exchange membrane fuel cells. Int. J. Hydrog. Energy.

[5] Xi Z.P., Zhu J.L., Tang H.P., Ao Q.B., Zhi H., Wang J.Y., Li C. (2011). Progress of application researches of porous fiber metals. Materials.

[6] Banhart J. (2001). Manufacture, characterisation and application of cellular metals and metal foams. Prog. Mater. Sci..

[7] Malheiro V.N., Skepper J.N., Brooks R.A., Markaki A.E. (2013). In vitro osteoblast response to ferritic stainless steel fiber networks for magneto-active layers on implants. J. Biomed. Mater. Res. A.

[8] Tang H.P., Zhang Z.D. (1997). Developmental states of porous metal materials. Rare Met. Mater. Eng..

[9] Zhou W., Tang Y., Pan M.P., Wei X.L., Xiang J.H. (2009). Experimental investigation on uniaxial tensile properties of high-porosity metal fiber sintered sheet. Mater. Sci. Eng. A Struct..

[10] García-Moreno F. (2016). Commercial applications of metal foams: Their properties and production. Materials.

[11] Liu P., He G., Wu L.H. (2009). Uniaxial tensile stress-strain behavior of entangled steel wire material. Mater. Sci. Eng. A Struct..

[12] Jiang G.F., Li Q.Y., Wang C.L. (2015). Fabrication of graded porous titanium-magnesium composite for load-bearing biomedical applications. Mater. Des..

[13] Ma C.Y., Ding W.F., Xu J.H., Fu Y.C. (2015). Influence of alumina bubble particles on microstructure and mechanical strength in porous Cu-Sn-Ti metals. Mater. Des..

[14] Cay H., Xu H.R., Li Q.Z. (2013). Mechanical behavior of porous magnesium/alumina composites with high strength and low density. Mater. Sci. Eng. A Struct..

[15] Rabiei A., O’Neill A.T. (2005). A study on processing of a composite metal foam via casting. Mater. Sci. Eng. A Struct..

[16] Vendra L., Rabiei A. (2010). Evaluation of modulus of elasticity of composite metal foams by experimental and numerical techniques. Mater. Sci. Eng. A Struct..

[17] Vendra L.J., Rabiei A. (2007). A study on aluminum-steel composite metal foam processed by casting. Mater. Sci. Eng. A Struct..

[18] Rabiei A., Vendra L.J. (2009). A comparison of composite metal foam’s properties and other comparable metal foams. Mater. Lett..

[19] Tang Y., Zhou R., Li H., Yuan W., Lu L.S. (2014). Experimental study on the tensile strength of a sintered porous metal composite. Mater. Sci. Eng. A Struct..

[20] Ackermann S., Scheffe J.R., Duss J., Steinfeld A. (2014). Morphological characterization and effective thermal conductivity of dual-scale reticulated porous structures. Materials.

[21] Fang C.B., Wan Z.P., Liu B., Lu L.S. (2014). A novel sintered stainless steel fiber felt with rough surface morphologies. Adv. Mater. Sci. Eng..

[22] Wu F., Zhou Z.Y., Duan L.Y., Xiao Z.Y. (2015). Processing, structural characterization and comparative studies on uniaxial tensile properties of a new type of porous twisted wire material. Materials.

[23] Wan Z.P., Tang Y., Liu Y.J., Liu W.Y. (2007). High efficient production of slim long metal fibers using bifurcating chip cutting. J. Mater. Process. Technol..

